# A pathogen-induced translational shift enhances plant disease resistance without obvious fitness costs

**DOI:** 10.1016/j.abiote.2026.100025

**Published:** 2026-02-03

**Authors:** Yuzhen Mei, Xiaofei Cheng, Yaqin Wang, Fangfang Li, Xueping Zhou

**Affiliations:** a State Key Laboratory of Rice Biology and Breeding, Institute of Biotechnology, Zhejiang University, Hangzhou, 310058, China; b State Key Laboratory for Biology of Plant Diseases and Insect Pests, Institute of Plant Protection, Chinese Academy of Agricultural Sciences, Beijing, 100193, China; c Key Laboratory of Germplasm Enhancement, Physiology and Ecology of Food Crops in Cold Region of Chinese Education Ministry, College of Plant Protection, Northeast Agricultural University, 150030, Harbin, China

**Keywords:** Specific translation leakage, Geminivirus C4, Disease resistance, Fitness costs

## Abstract

Exploring immune mechanisms in plants opens new avenues for engineering disease resistance in crops. Approaches such as the ectopic expression of resistance genes are frequently associated with fitness costs, rendering the resulting germplasm less desirable for agricultural applications. Inducible production of resistance factors is one potential workaround for this problem. In this study, we determined that the 5ʹ untranslated region (5ʹ UTR) of Arabidopsis (*Arabidopsis thaliana*) *PI4KIII β1* undergoes a translational shift that alters its translation initiation site upon the activation of immunity. Based on this discovery, we developed a *PI4KIII β1* 5ʹ UTR cassette to specifically regulate the expression of the hypersensitive induced reaction gene *HIR1*, which encodes a cell death-inducing protein. Under normal conditions, ribosomes initiated translation from the first start codon in the *PI4KIII β1* 5ʹ UTR cassette; however, upon pathogen challenge, the ribosomes bypassed this site and instead initiated translation from a downstream start codon, enabling *HIR1* expression. Transgenic *Arabidopsis* plants harboring the *PI4KIII β1* 5ʹ UTR cassette driving *HIR1* exhibited enhanced disease resistance without detectable changes in key agricultural traits. Therefore, precisely regulating translation initiation through leaky ribosome scanning represents a promising strategy for developing pathogen-resistant crops without obvious fitness costs.

## 1 Introduction

Engineering plant disease resistance by expressing resistance factors, such as pathogen-associated molecular pattern (PAMP) receptors, often results in decreased plant fitness and decreased yields, referred to as the defense-yield tradeoff. Constitutive expression of resistance factors exacerbates this problem. Therefore, one approach to mitigating effects on yields is to express resistance factors from inducible promoters. For example, pathogen-inducible Ta-Lr34res (an ABC transporter) expression in heterologous barley confers disease resistance without negative pleiotropic effects [1].

Modulation of translational initiation plays a key role in regulating gene expression. In eukaryotes, most messenger RNAs (mRNAs) are translated in a cap-dependent manner, with the 43S preinitiation complex scanning from the 5ʹ cap and initiating translation at the first start codon [[2], [3], [4], [5], [6]]. However, the presence of upstream start codons (uAUGs), which are recognized as initiation codons, leads to the abortion of translation initiation at the AUG of the main open reading frame (mORF), providing alternative translation start sites [[7], [8], [9], [10]]. These uAUGs allow the preinitiation complex to start translation before reaching the main AUG (mAUG), diversifying the proteome by producing different translation products. Translational shifts driven by leaky ribosome scanning enable the production of diverse protein isoforms, facilitating the adaptation of organisms to changing conditions [[11], [12], [13], [14], [15]].

In general, uAUGs inhibit translation from downstream mAUGs [8,[16], [17], [18]], which is critical for controlling the production of specific proteins under normal conditions [[19], [20], [21]]. For example, mammalian and plant viruses use both canonical and noncanonical translation initiation sites to produce diverse viral effectors during infection [[22], [23], [24], [25]].

Alternative protein translation mediated by uAUGs occurs widely in genes involved in plant immunity and pathogen virulence [15,[24], [25], [26]]. For example, the translation of TL1-BINDING TRANSCRIPTION FACTOR 1 (TBF1), a key immunity-related transcription factor with a substantial effect on downstream defense responses, is normally suppressed by two uORFs in its 5ʹ leader sequence under normal growth conditions but is activated upon immune priming [27,28]. Indeed, transcripts with immunity-induced translation are enriched in uORFs that are selectively translated due to the presence of hairpins immediately downstream of uAUGs under normal conditions [14]. In rice (*Oryza sativa*), alternative translation initiation from both the mORF and downstream in-frame ORF of *OsWRKY7* generates two protein isoforms with different stabilities, which is essential for establishing effective basal defense [15].

PI4KIII β1, a critical component of lipid signaling and a negative regulator of the activation of the salicylic acid (SA) signaling pathway in Arabidopsis (*Arabidopsis thaliana*), possesses two in-frame neighboring start codons in its mRNA, which can produce two distinct translation products differing in their two N-terminal amino acids. Notably, the 5ʹ untranslated region (5ʹ UTR) of *PI4KIII β1* plays a critical role in the translational shift in response to pathogen attack, leading to a switch in the translation initiation site upon immune priming.

Hypersensitive induced reaction genes (*HIR*) encode a cell death-inducing protein, it plays important roles in cell proliferation, ion channel regulation, and cell death [29,30]. Induction of *HIR* genes occurs upon pathogen attack, including bacteria, fungi and viruses and accumulation of HIR proteins can induce host cell death and disease resistance responses [29,[31], [32], [33], [34], [35], [36]].

In the current study, we established a *PI4KIII β1* 5ʹ UTR–containing reporter system responsive to PAMP treatment and developed a *PI4KIII β1* 5ʹ UTR cassette consisting of the immunity-inducible translational shift element and additional translation initiation regulatory sequences. We used this cassette to direct the specific expression of *HIR1* in transgenic *Arabidopsis* plants. This cassette conferred broad-spectrum disease resistance without compromising fitness. Regulating translation initiation for the targeted control of defense-related protein production might represent an effective, sustainable strategy to generate disease-resistant crops.

## 2 Results

### 2.1 PI4KIII β1 *undergoes alternative translation initiation from a neighboring in-frame start codon in a PAMP-dependent manner*

**Fig. 1:**
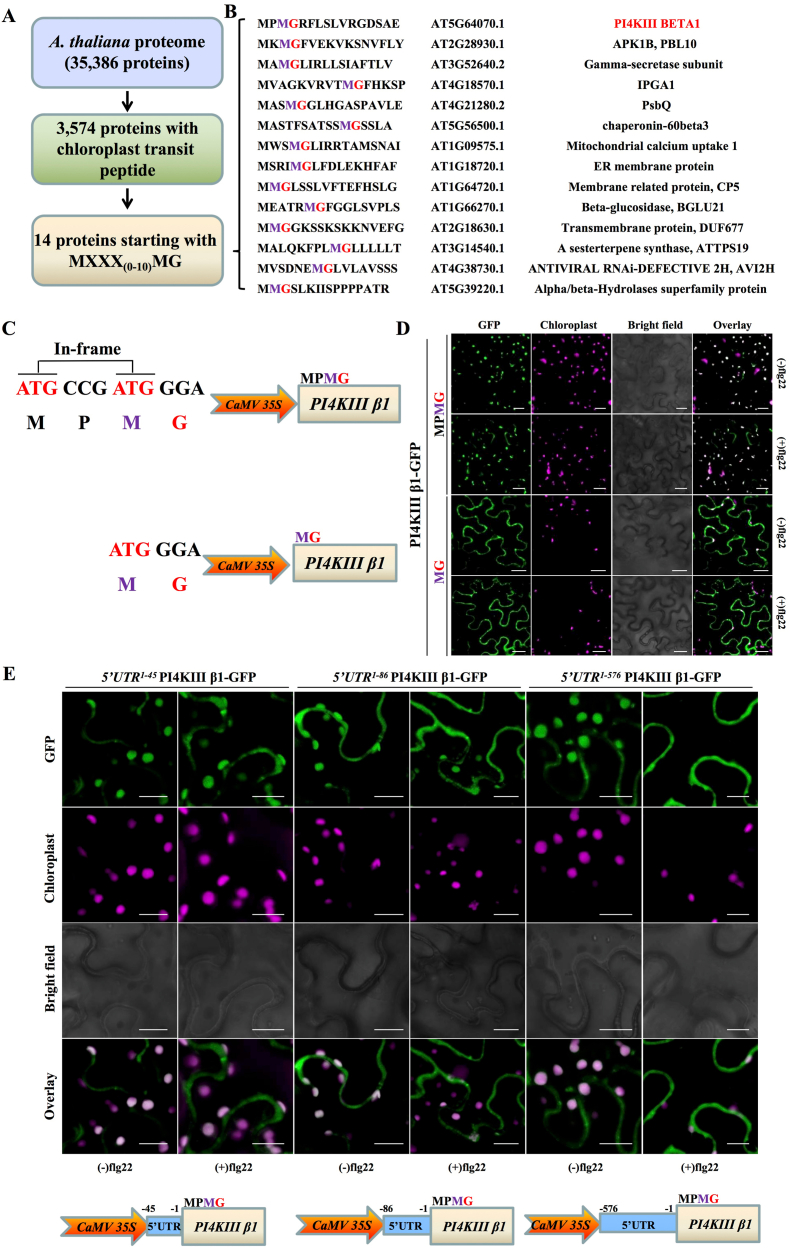
The 5′ untranslated region (5′ UTR) of *PI4KIII β1* plays an important role in regulating the translational shift upon pathogen-associated molecular pattern (PAMP) treatment. **A** Flow diagram illustrating the number of proteins encoded by the *Arabidopsis thaliana* genome containing both a predicted chloroplast transit peptide (cTP) and an in-frame start codon similar to those in geminivirus *AC4/C4*. **B** Accession numbers and descriptions of *A. thaliana* candidate proteins with a predicted cTP and an in-frame start codon. **C** Nucleotide sequences of *PI4KIII β1* containing two potential in-frame start codons. The encoded amino acid sequences of two PI4KIII β1 isoforms are shown. **D** Subcellular localizations of two PI4KIII β1 variants fused to GFP in *N. benthamiana* leaf epidermal cells following treatment with 1 μM flg22 at 24 h post-infiltration (hpi). Scale bar = 50 μm. **E** The 5ʹ UTR of *PI4KIII β1* plays an important role in start codon selection upon PAMP treatment. The subcellular distribution of PI4KIII β1 produced by using full-length and truncated versions of the 5ʹ UTR of *PI4KIII β1* in *N. benthamiana* leaves was observed under a confocal microscope. A diagram of *PI4KIII β1* expression driven by full-length and truncated versions of the 5ʹ UTR of *PI4KIII β1* is shown in the lower panel. Scale bar = 50 μm.

Geminivirus *AC4/C4* possesses two in-frame start codons that produce two distinct translation products from a single ORF: chloroplast-localized AC4/C4, which contains a chloroplast transit peptide (cTP); and membrane-associated AC4/C4, which harbors an N-terminal myristoylation motif [25]. To investigate whether a similar translation regulatory mechanism also exists in plants, we screened the *Arabidopsis* proteome for proteins containing a putative cTP with an N-myristoylation motif encoded by a gene with an in-frame start codon. We identified 14 candidate proteins (Fig. 1A and B). Notably, some of these proteins are defense-related, such as type III phosphatidylinositol-4-kinase subunit beta 1 (PI4KIII β1) (Fig. S1). PI4KIIIs play important roles in regulating plant immunity; SA signaling is constitutively activated in the *pi4kIII β1β2* mutant [37]. Consistently, *ICS1* and *PR-1* were significantly upregulated in the *pi4kIII β1* mutant compared to wild-type plants (Fig. S2).

The presence of two potential in-frame start codons in *PI4KIII β1* raised the possibility that two distinct translation products differing in their two N-terminal amino acids might be translated from a single mRNA. To explore this hypothesis, we transiently expressed the two potential *PI4KIII β1* isoforms driven by the cauliflower mosaic virus (CaMV) 35S promoter, to produce C-terminal GFP-tagged recombinant proteins in *Nicotiana benthamiana* epidermal cells. Confocal microscopy at 48 h post-infiltration (hpi) revealed that the longer isoform localized primarily to chloroplasts, whereas the shorter isoform, containing a conserved N-myristoylation motif, was detected at the cell periphery (Fig. 1C and D), confirming divergent subcellular targeting of the two isoforms. Treatment with flg22, a bacterial flagellin-derived PAMP [38,39], did not alter the subcellular distribution of these proteins (Fig. 1D and S3). To determine whether N-myristoylation of the shorter isoform of PI4KIII β1 is a functional modification, we mutated the Gly2 to an Ala residue to produce the non-myristoylatable mutant PI4KIII β1^G2A^. Confocal microscopy showed that much more PI4KIII β1^G2A^-GFP than PI4KIII β1-GFP accumulated in chloroplasts (Fig. S4). These results suggest that N-myristoylation of PI4KIII β1 overrides its cTP function and is critical for the membrane association of PI4KIII β1.

To identify the *cis*-element that determines the translational shift of *PI4KIII β1*, we fused three different fragments of the 5ʹ UTR (−1 to −45 nt, −1 to −86 nt, or the full-length 5ʹ UTR [−1 to −576 nt]) to the *PI4KIII β1* coding sequence and expressed these constructs in *N. benthamiana* leaves under the control of the CaMV 35S promoter. At 24 hpi, we treated infiltrated leaves with 1 μM flg22 and analyzed them by confocal microscopy. Flg22 treatment induced a significant shift in subcellular localization for the construct containing the full-length 5ʹ UTR but not for the two truncated versions, with clear PI4KIII β1-GFP accumulation at the plasma membrane (PM) (Fig. 1E and S5).

To determine whether the shift in the chloroplast/PM PI4KIII β1-GFP ratio was due to differential targeting following *de novo* synthesis and not to the physical re-localization of the protein, we treated *N. benthamiana* leaves with the translation inhibitor cycloheximide (CHX), a chemical commonly used to abolish newly synthesized isoforms that only slightly affects the abundance or function of the protein translocation machinery [40]. CHX treatment abolished this change in localization (Fig. S6), supporting the idea that the change in subcellular localization resulted from a newly synthesized isoform rather than from the re-localization of pre-existing proteins.

To determine whether the alternative AUG usage would be preserved under the native promoter of *PI4KIII β1*, we inserted a 21-nucleotide (encoding 7 × His) spacer between the two in-frame start codons of the *PI4KIII β1* coding sequence to construct _*MP-7His-*_*PI4KIII β1-GFP*. We also created a frameshift mutation by deleting a cytosine (_*MP-7His(-C)-*_*PI4KIII β1-GFP*). We then examined alternative AUG usage under the control of the *PI4KIII β1* native promoter by immunoblotting using anti-GFP and anti-His tag antibodies (Fig. S7). The results support the use of alternative AUGs under the control of the *PI4KIII β1* native promoter upon flg22 treatment *in planta*. Taken together, these results suggest that the 5ʹ UTR of *PI4KIII β1* mediates a translational shift upon immune activation.

### *2.2* *Establishing the* PI4KIII β1 5ʹ UTR *reporter system*

To further investigate the translational regulation mediated by the 5ʹ UTR of *PI4KIII β1*, we developed a reporter system using a nuclear localization signal (NLS) and membrane-targeting sequences (MTS) to visualize the products of the translational shift. We fused either the N-myristoylation motif from NtCDPK1 (termed NmM) or an NLS to GFP at each terminus (N or C) or at both termini and transiently expressed these constructs in H2B-RFP (full-length red fluorescent protein fused to the C terminus of histone 2B) transgenic *N. benthamiana* plants under the control of the CaMV 35S promoter. Confocal microscopy at 2 dpi confirmed that the NLS directed GFP to the nucleus, whereas MTS targeted GFP to the PM; when both signals were present, GFP localized to both compartments (Fig. S8). To confirm that the membrane localization of NmM-GFP-NLS depended on N-myristoylation, we generated two non-myristoylatable versions of this protein (NmM_G2A_-GFP-NLS and _MP_NmM-GFP-NLS) by mutating the N-terminal glycine residue (Gly2) or adding two amino acids (MP) to the N terminus of the NmM-GFP-NLS coding sequence. As expected, these mutant proteins localized exclusively to the nucleus (Fig. S8), confirming the critical role of N-myristoylation in membrane targeting.

**Fig. 2:**
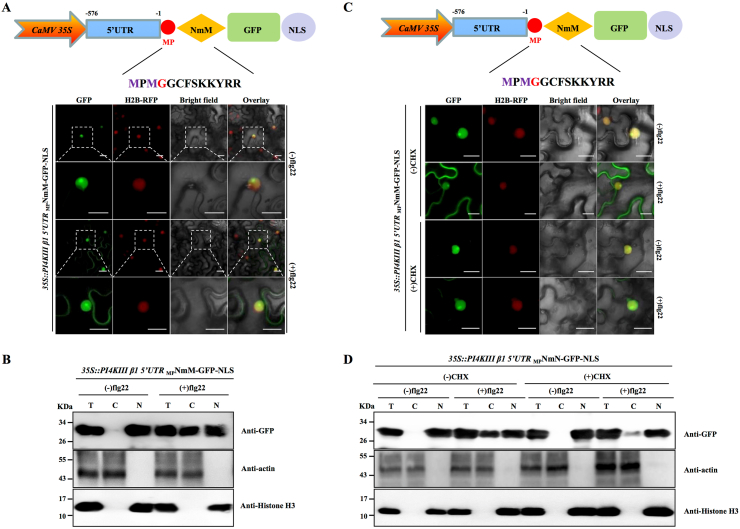
Analysis of the translational shift by the *PI4KIII β1* 5ʹ UTR upon flg22 treatment. **A** Confocal observation of the subcellular distribution of _MP_NmM-GFP-NLS produced by using the CaMV 35S promoter and the full-length 5ʹ UTR of *PI4KIII β1* in epidermal cells of H2B-RFP transgenic *N. benthamiana* plants following treatment with 1 μM flg22 at 24 hpi. Scale bar = 50 μm. **B** Nuclear–cytoplasmic fractionation analysis of the altered cellular distribution of _MP_NmM-GFP-NLS in the absence or presence of flg22. Immunoblot analysis was conducted with antibodies specific to the indicated proteins. NbActin was used as a marker for the cytoplasmic fraction, and histone H3 was used as a marker for the nuclear fraction. T, C, and N indicate total, cytoplasmic, and nuclear extracts, respectively. **C** Subcellular localization of _MP_NmM-GFP-NLS produced by using the CaMV 35S promoter and the full-length 5ʹ UTR of *PI4KIII β1* in epidermal cells of H2B-RFP transgenic *N. benthamiana* plants following treatment with 1 μM flg22 in the presence of CHX. Leaves of H2B-RFP transgenic *N. benthamiana* plants transiently expressing _MP_NmM-GFP-NLS were treated with 1 μM flg22 (24 h post-treatment) in the presence of CHX (50 mM, 2 h prior to flg22 treatment) before confocal microscopy. Scale bar = 50 μm. **D** Nuclear–cytoplasmic fractionation analysis of the altered cellular distribution of _MP_NmM-GFP-NLS in tissues treated with flg22 in the presence of CHX. Immunoblot analysis was conducted with antibodies specific to the indicated proteins. Actin was used as a marker for the cytoplasmic fraction, and histone H3 was used as a marker for the nuclear fraction. T, C, and N indicate total, cytoplasmic, and nuclear extracts, respectively.

To examine the translational regulation mediated by the 5ʹ UTR of *PI4KIII β1*, we fused this regulatory element to _MP_NmM-GFP-NLS and introduced the construct into *N. benthamiana* leaves. Before flg22 treatment, the GFP signal exclusively localized to the nucleus, whereas after flg22 treatment, a cytosolic GFP signal became apparent (Fig. 2A), suggesting that the *PI4KIII β1* 5ʹ UTR undergoes a translational shift upon immune activation, producing both nucleus-localized _MP_NmM-GFP-NLS and membrane-associated NmM-GFP-NLS isoforms.

To confirm these observations, we performed a nuclear–cytoplasmic fractionation assay. Using actin and histone H3 as cell compartment-specific markers, we detected GFP in the cytoplasmic fractions after flg22 treatment (Fig. 2B), confirming that the 5ʹ UTR of *PI4KIII β1* enables a translational shift upon immune activation. Consistent with these results, GFP was also detected in the cytoplasmic fractions after chitin treatment (Fig. S9). These results indicate that the translational shift mediated by the 5ʹ UTR of *PI4KIII β1* responds to PAMPs and has great potential for widespread applicability.

To exclude the possibility that _MP_NmM-GFP-NLS moved from the nucleus to the cytosol upon flg22 treatment, we treated the leaves of H2B-RFP transgenic *N. benthamiana* plants transiently expressing _MP_NmM-GFP-NLS with the *PI4KIII β1* full-length 5ʹ UTR with 50 mM CHX for 2 h, followed by flg22 treatment. Both confocal microscopy and nuclear–cytoplasmic fractionation assays demonstrated the persistent nuclear localization of GFP under these conditions, whereas the cytoplasmic signal detected in the presence of flg22 significantly decreased in the presence of CHX (Fig. 2C and D). Collectively, these results suggest that membrane-associated GFP originated from *de novo* synthesis of NmM-GFP-NLS rather than the translocation of pre-existing _MP_NmM-GFP-NLS.

**Fig. 3:**
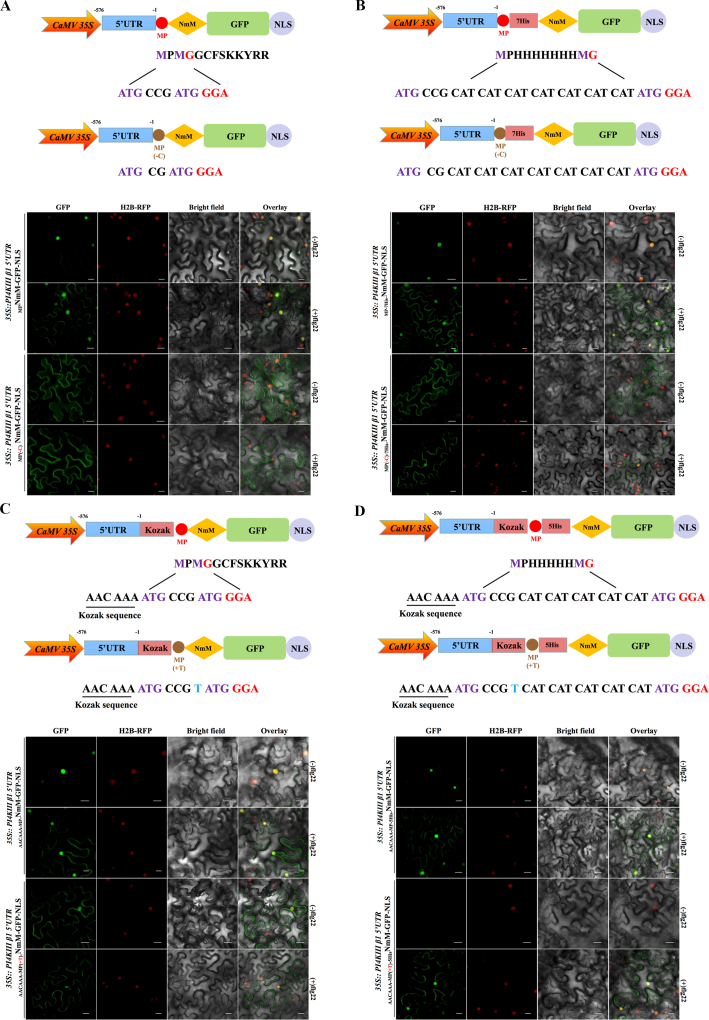
Establishment of a *PI4KIII β1* 5ʹ untranslated region (5ʹ UTR)–containing reporter system to detect the translational shift. **A** Analysis of the translational shift by the *PI4KIII β1* 5ʹ UTR after deleting a nucleotide between two in-frame start codons. Upper panel shows diagrams of *PI4KIII β1* 5ʹ UTR_*MP*_*NmM-GFP-NLS* and the mutant. Lower panel shows confocal micrographs of GFP signals in *N. benthamiana* leaf epidermal cells following treatment with 1 μM flg22 at 24 hpi. Scale bar = 50 μm. **B** Analysis of the translational shift by the *PI4KIII β1* 5ʹ UTR after increasing the distance between two in-frame start codons. Upper panel shows diagrams of *PI4KIII β1* 5ʹ UTR_*MP-7His*_*NmM-GFP-NLS* and the mutant. Lower panel shows confocal micrographs of GFP signals in *N. benthamiana* following treatment with 1 μM flg22 at 24 hpi. Scale bar = 50 μm. **C** Analysis of the translational shift by the 5ʹ UTR of *PI4KIII β1* after introducing a Kozak sequence and inserting a nucleotide between two in-frame start codons. Upper panel shows diagrams of *PI4KIII β1* 5ʹ UTR-_*Kozak-MP*_*NmM-GFP-NLS* and the mutant. Lower panel shows confocal micrographs of GFP signals in *N. benthamiana* following treatment with 1 μM flg22 at 24 hpi. Scale bar = 50 μm. **D** Analysis of the translational shift by the 5ʹ UTR of *PI4KIII β1* after introducing a Kozak sequence and increasing the distance between two in-frame start codons. Upper panel shows diagrams of *PI4KIII β1* 5ʹ UTR-_*Kozak-MP-5His-*_*NmM-GFP-NLS* and the mutant. Lower panel shows confocal micrographs of GFP signals in *N. benthamiana* following treatment with 1 μM flg22 at 24 hpi. Scale bar = 50 μm.

To develop a protein production system specifically responsive to PAMP recognition, we modified the _*MP*_*NmM-GFP-NLS* construct containing the *PI4KIII β1* 5ʹ UTR by deleting a cytosine between the two in-frame start codons to create _*MP(-C)*_*NmM-GFP-NLS*. This frameshift was designed to disrupt the translation product from the first start codon; however, GFP signal persisted in epidermal cells regardless of flg22 treatment (Fig. 3A), indicating that leaky translation initiation could also occur at the start codon of *NmM-GFP-NLS* ORF without activation of the immune response. To inhibit leaky translation, we inserted a 21-nucleotide (encoding 7 × His) spacer between the two in-frame start codons to construct _*MP-7His*_*NmM-GFP-NLS*. We also create a frameshift mutation by deleting a cytosine (_*MP(-C)-7His*_*NmM-GFP-NLS*). Although this strategy reduced background expression, slight GFP signal remained detectable before flg22 treatment, as revealed by confocal microscopy (Fig. 3B).

To enhance the recognition of the first start codon, we introduced a Kozak sequence upstream of the _*MP*_*NmM-GFP-NLS* coding sequence (_*Kozak-MP*_*NmM-GFP-NLS*) and created another frameshift variant by adding a thymine between the two in-frame start codons (_*Kozak-MP(+T)*_*NmM-GFP-NLS*). However, a translational shift was also detected in *N. benthamiana* epidermal cells containing the _*Kozak-MP(+C)*_*NmM-GFP-NLS* sequence without flg22 treatment (Fig. 3C). Through iterative optimization, we developed a construct containing a Kozak sequence upstream of the first ATG, a 15-nucleotide spacer encoding five His residues between the two in-frame start codons (_*Kozak-MP-5His*_*NmM-GFP-NLS*), and a frameshift variant created by adding a thymine insertion between the two in-frame start codons (_*Kozak-MP(+T)-5His*_*NmM-GFP-NLS*). Cells expressing _*Kozak-MP(+T)-5His*_*NmM-GFP-NLS* under *PI4KIII β1* 5ʹ UTR had no detectable GFP signals under normal conditions but clear signals after flg22 treatment, as revealed by confocal microscopy (Fig. 3D). This indicates that this construct showed strict PAMP-inducible expression.

To further validate the utility of this construct, we replaced *GFP* with a luciferase reporter and produced transgenic *Arabidopsis* plants. Following flg22 and chitin treatment, luciferase activity was significantly induced in the transgenic plants compared to the water controls upon PAMP perception (Fig. S10), demonstrating that the *PI4KIII β1* 5′ UTR can be modified for precisely controlled translation of target proteins specifically during immune responses.

### 2.3 Translational shift–mediated accumulation of a resistance protein upon PAMP recognition does not affect plant growth

The activation of immunity in plants involves redirecting resources from growth-related activities to defense, which often results in fitness costs. The *PI4KIII β1* 5ʹ UTR–mediated translational shift offers an opportunity to precisely control defense protein translation, which could minimize fitness penalties when engineering plants with increased disease resistance. To test our hypothesis, we selected the candidate gene *HIR1* as a proof of concept. Although overexpressing *HIR* enhanced resistance to various pathogens, it also led to autoimmune phenotypes, such as spontaneous necrotic lesion formation (Fig. S11). We thus evaluated the capacity of the *PI4KIII β1* 5ʹ UTR to control HIR1 production with either the 21-nucleotide spacer, Kozak sequence, or both.

**Fig. 4:**
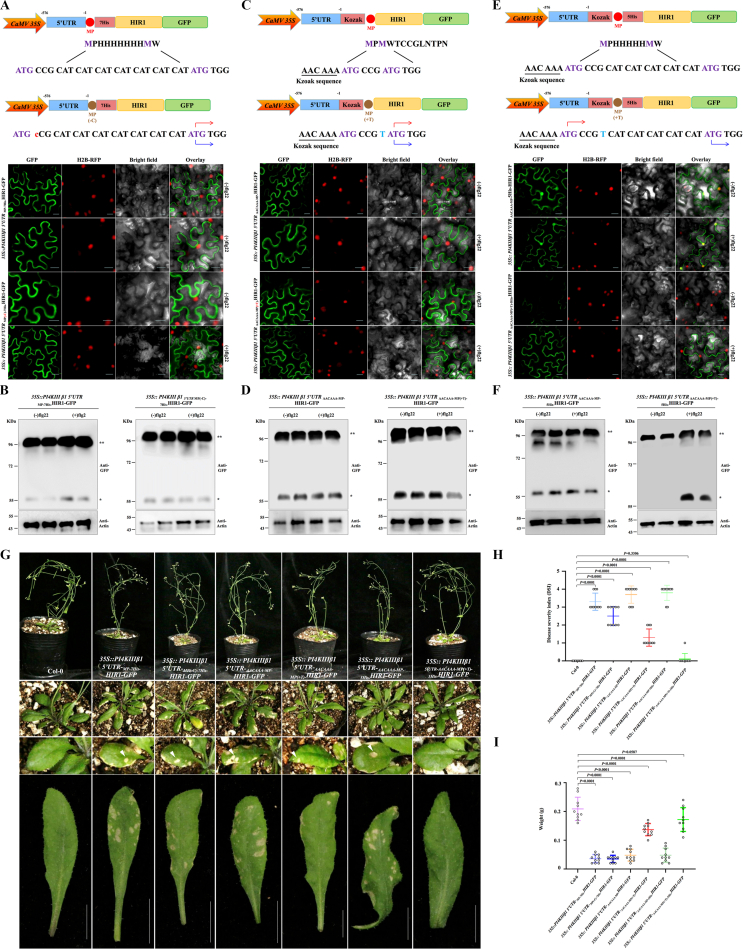
*PI4KIII β1* 5ʹ UTR cassette–mediated production of a resistance protein via a translational shift has no obvious fitness costs. **A** Analysis of resistance gene expression mediated by the *PI4KIII β1* 5ʹ UTR cassette containing a 21-bp sequence insertion between two in-frame start codons. Upper panel shows diagrams of *PI4KIII β1* 5ʹ UTR-_*MP-7His-*_*HIR1-GFP* and the mutant. The red arrow indicates translation initiation under normal conditions, and the blue arrow denotes translation initiation under immunity-inducing conditions. The lower panel shows confocal micrographs of HIR1-GFP signals in *N. benthamiana* leaf epidermal cells following treatment with 1 μM flg22 at 24 hpi. Scale bar = 50 μm. **B** Immunoblot analysis of HIR1-GFP accumulation mediated by the *PI4KIII β1* 5ʹ UTR cassette shown in Fig. 4A following treatment with 1 μM flg22 at 24 hpi. NbActin was used as the loading control. ∗, HIR1 monomer bands; ∗∗, HIR1 dimer bands. **C** Analysis of resistance gene expression mediated by the *PI4KIII β1* 5ʹ UTR cassette containing a Kozak sequence. Upper panel shows diagrams of *PI4KIII β1* 5ʹ UTR-_*Kozak-MP-*_*HIR1-GFP* and the mutant. The red arrow indicates translation initiation under normal conditions, and the blue arrow denotes translation initiation under immunity-inducing conditions. Lower panel shows confocal micrographs of HIR1-GFP signals in *N. benthamiana* following treatment with 1 μM flg22 at 24 hpi. Scale bar = 50 μm. **D** Immunoblot analysis of HIR1-GFP accumulation mediated by the *PI4KIII β1* 5ʹ UTR cassette shown in Fig. 4C following treatment with 1 μM flg22 at 24 hpi. NbActin was used as the loading control. ∗, HIR1 monomer bands; ∗∗, HIR1 dimer bands. **E** Analysis of the translational shift by the *PI4KIII β1* 5ʹ UTR after introducing a Kozak sequence and inserting a 15-bp sequence between two in-frame start codons. Upper panel shows diagrams of *PI4KIII β1* 5ʹ UTR-_*Kozak-MP-5His-*_*HIR1-GFP* and the mutant. The red arrow indicates translation initiation under normal conditions, and the blue arrow denotes translation initiation under immunity-inducing conditions. Lower panel shows confocal micrographs of HIR1-GFP signals in *N. benthamiana* following treatment with 1 μM flg22 at 24 hpi. Scale bar = 50 μm. **F** Immunoblot analysis of HIR1-GFP accumulation mediated by the *PI4KIII β1* 5ʹ UTR cassette shown in Fig. 4E following treatment with 1 μM flg22 at 24 hpi. NbActin was used as the loading control. ∗, HIR1 monomer bands; ∗∗, HIR1 dimer bands. **G** Phenotypes of wild-type and transgenic *Arabidopsis* plants overexpressing *HIR1* mediated by the *PI4KIII β1* 5ʹ UTR cassette. White arrowheads indicate lesions caused by *HIR1* overexpression. Lower panel shows necrotic spots induced by the auto-activation of immunity in the leaves of transgenic *Arabidopsis* plants. Photographs were taken at 45 days post-germination (dpg). Scale bar = 1 cm. **H** Severity of necrosis induced by the auto-activation of immunity in leaves of wild-type and transgenic *Arabidopsis* plants. Error bars show standard deviation for 10 individual samples in two independent transgenic lines. Significant differences were analyzed by two-sided, unpaired Student's *t*-test. Data are presented as mean values ± SD. Individual *P* values between two groups are shown at the top. **I** Total seed weight of wild-type and transgenic *A. thaliana* plants. Error bars show standard deviation for 10 individual samples in two independent transgenic lines. Significant differences were analyzed by two-sided, unpaired Student's *t*-test. Data are presented as mean values ± SD. Individual *P* values between two groups are shown at the top.

Confocal microscopy and immunoblot analysis showed that the *PI4KIII β1* 5ʹ UTR–mediated translational shift specifically regulated HIR1 translation in *N. benthamiana* plants harboring the _*Kozak-MP(+T)-5His*_*HIR1-GFP* construct upon flg22 treatment (Fig. 4A–F). These results support the potential of this cassette to tightly control the expression of resistance proteins to engineer disease resistance. We then produced transgenic *Arabidopsis* plants expressing *HIR1* under the control of the *PI4KIII β1* 5ʹ UTR and either the 21-nucleotide spacer, Kozak sequence, or both. To confirm that the *PI4KIII β1* 5ʹ UTR regulates *HIR1* expression through translational control, specifically by regulating the initiation site, we conducted RT-qPCR of *HIR1* mRNA levels in stable transgenic plants treated (or not) with flg22. *HIR1* transcription was not upregulated upon flg22 treatment (Fig. S12). Although constitutive *HIR1* overexpression caused severe leaf lesions, the expression of _*Kozak-MP(+T)-5His*_*HIR1-GFP*, but not other constructs, from the *PI4KIII β1* 5ʹ UTR cassette allowed normal growth, with no visible necrotic spots on leaves and no change in seed weight per plant (Fig. 4G–I). These results indicate that the *PI4KIII β1* 5ʹ UTR can eliminate the fitness costs typically associated with resistance protein production.

### 2.4 A resistance protein is overproduced via a translational shift upon PAMP detection, conferring broad resistance to pathogens

**Fig. 5:**
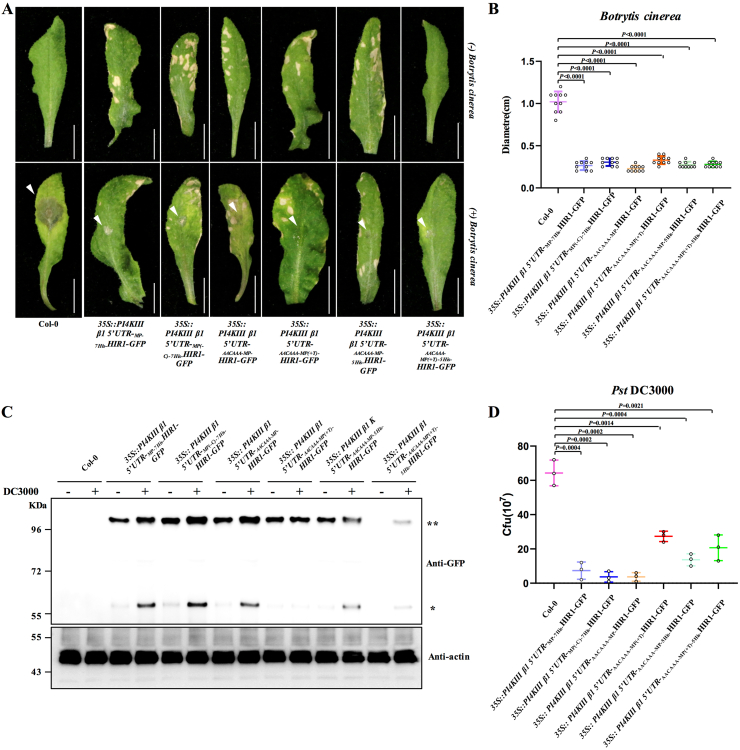
*PI4KIII β1* 5ʹ UTR cassette–mediated production of a resistance protein confers broad resistance to pathogens without associated growth defects. **A** Representative phenotypes and symptoms observed in wild-type and transgenic *Arabidopsis* plants. White arrowheads indicate leaf lesions caused by *B. cinerea*. **B** Quantification of leaf lesion diameter in wild-type and transgenic *A. thaliana* plants. Error bars show standard deviation for 10 individual samples in two independent transgenic lines. Significant differences were analyzed by two-sided, unpaired Student's *t*-test. Data are presented as mean values ± SD. Individual *P* values between two groups are shown at the top. **C** Immunoblot analysis of HIR1 accumulation in wild-type and transgenic *A. thaliana* plants after inoculation with *Pseudomonas syringae* pv. *tomato* DC3000 (*Pst* DC3000). ∗, HIR1 monomer bands; ∗∗, HIR1 dimer bands. **D***PI4KIII β1* 5ʹ UTR cassette–mediated specific expression of *HIR1* enhances resistance to DC3000. Significant differences were analyzed by two-sided, unpaired Student's *t*-test. Data are presented as mean values ± SD. Individual *P* values between two groups are shown at the top.

To investigate whether these transgenic *Arabidopsis* plants exhibited enhanced pathogen resistance, we challenged them with the plant pathogenic necrotrophic fungus *Botrytis cinerea*. Although all transgenic plants displayed increased resistance to *B*. *cinerea*, only plants harboring _*Kozak-MP(+T)-5His*_*NmM-HIR1-GFP* driven by the *PI4KIII β1* 5ʹ UTR avoided the associated fitness costs, such as leaf lesions (Fig. 5A and B). To assess whether this system confers resistance to other pathogens, we inoculated these transgenic plants with the hemibiotrophic pathogenic bacterium *Pseudomonas syringae* pv. *tomato* (*Pst* DC3000). Analysis of HIR1 accumulation and bacterial colony-forming units (cfu) at 72 hpi revealed that transgenic *Arabidopsis* plants with the *PI4KIII β1* 5ʹ UTR cassette had higher HIR1 protein levels and exhibited enhanced resistance during DC3000 infection (Fig. 5C and D).

**Fig. 6:**
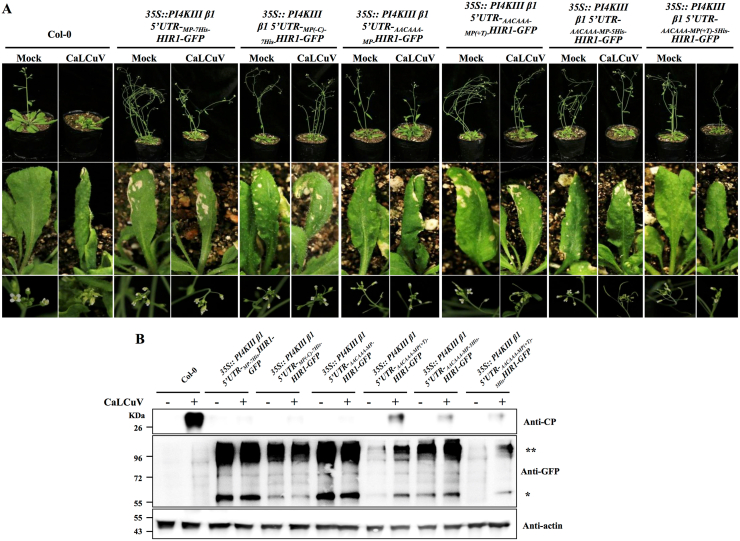
*PI4KIII β1* 5ʹ UTR cassette–mediated specific translation of a resistance protein confers resistance to a plant virus without fitness costs. **A** Representative viral symptoms observed in wild-type and transgenic *Arabidopsis thaliana* plants. Upper, middle, and bottom panels show the phenotypes of whole plants, leaves, and inflorescences, respectively, of wild-type and transgenic *A. thaliana* plants infected by cabbage leaf curl virus (CaLCuV) at 14 dpi. **B** Immunoblot analysis of the accumulation of HIR1 and CaLCuV coat protein (CP) in wild-type and transgenic *A. thaliana* plants after inoculation with CaLCuV. Actin2 was used as loading control. ∗, HIR1 monomer bands; ∗∗, HIR1 dimer bands.

Finally, we examined the resistance of the transgenic plants to cabbage leaf curl virus (CaLCuV). Consistent with previous results, transgenic *Arabidopsis* plants expressing _*Kozak-MP(+T)-5His*_*NmM-HIR1-GFP* under the control of the *PI4KIII β1* 5ʹ UTR cassette displayed enhanced resistance to CaLCuV without growth penalties (Fig. 6A and B). These findings demonstrate that the *PI4KIII β1* 5ʹ UTR cassette mediates tightly regulated resistance protein production exclusively during pathogen infection and eliminates fitness trade-offs. Therefore, this system holds great promise for engineering disease resistance in plants.

## 3 Discussion

Traditionally, a uORF is defined as an ORF containing both a start codon and an in-frame stop codon located upstream of the mORF in the 5ʹ UTR of an mRNA. uORFs play crucial roles in translational regulation, with their translation being strictly controlled by hairpins immediately downstream of uAUGs [14,28]. Therefore, the structure of mRNA dynamically regulates the selection of start codons, and the remodeling of RNA structure represents a fundamental mechanism for translational reprogramming. In the current study, we demonstrated that the 5ʹ UTR of *PI4KIII β1* regulates start codon selection in response to biotic stress. However, no uORF is predicted in this sequence, and only two in-frame start codons are present, which are separated by only three nucleotides, which is insufficient for hairpin formation. These observations strongly suggest the existence of yet-to-be-described mechanisms governing the translation of *PI4KIII β1*. Systemic analysis of the 5ʹ UTRs of genes containing similar in-frame start codons could provide new insights into the molecular mechanisms of start codon selection and condition-dependent translation initiation.

In eukaryotes, translation initiation begins with the loading of the small ribosomal subunit onto the 5ʹ cap of the mRNA, followed by ribosome scanning in the 3ʹ direction. When the ribosomal subunit encounters an AUG, the translation machinery assembles and protein synthesis begins [41]. The production of two distinct proteins from a single mRNA typically occurs through context-dependent leaky scanning. Both the 5ʹ UTR and downstream RNA secondary structure can influence translation initiation [11,12,42]. Our findings support this idea and further reveal that the 5ʹ UTR of *PI4KIII β1* plays a critical role in start codon selection during immunity priming. However, the precise molecular mechanism remains elusive. RNA binding proteins (RBPs) play crucial roles in almost all cellular processes, and may coordinate mRNA translation by binding to specific sequence motifs and via combinatorial interactions with other RBPs [43]. We suggest that specific RBPs contribute to *PI4KIII β1* 5ʹ UTR–dependent start codon selection during defense activation. Further studies are needed to test this hypothesis.

The translational shift mediated by the *PI4KIII β1* 5ʹ UTR cassette is sensitive to the activation of PAMP-triggered immunity and has great potential for enhancing plant immunity without obvious fitness costs. Although transient expression of *HIR1* mediated by the *PI4KIII β1* 5ʹ UTR cassette resulted in detectable HIR1 accumulation in *N. benthamiana* even in the absence of flg22 treatment, transgenic *Arabidopsis* plants harboring this construct exhibited strict control of *HIR1* expression. This discrepancy between the results of transient expression in *N. benthamiana* and stable expression in *Arabidopsis* may result either from the activation of *Agrobacterium*-mediated defense or from differences between the plant species.

Although uAUGs have a less favorable Kozak sequence context than mAUGs, emerging evidence suggests that abiotic and biotic stress conditions can induce a translational shift by initiating translation from uAUGs [[44], [45], [46]]. The finding that the translational shift mediated by the *PI4KIII β1* 5ʹ UTR cassette is induced upon PAMP treatment is reminiscent of findings from previous studies in which ORFs were changed, uORF types were switched, and dual-initiation mechanisms were examined. Translation of the RBP FLOWERING CONTROL LOCUS A (FCA) can occur from a noncanonical CUG codon upstream of the first in-frame AUG; the upstream flanking sequence is essential for the selection of the translation initiation site [47]. uORFs in the 5′ leader from *Arabidopsis* *TBF1* were edited by CRISPR-Cas9 to generate uORF variants with different translation efficiencies to optimize the translational control of disease-related genes. The use of edited uORF variants to boost or dampen the translation of resistance-associated genes provided broad-spectrum disease resistance in crops with minimal fitness costs [48]. Further efforts to produce a translational shift that is responsive to a specific abiotic or biotic stress by integrating several strategies should help accelerate the breeding of disease-resistant crops.

Pathogen attack triggers host immune responses that are otherwise tightly suppressed through multi-level regulation. Most studies performed to date have focused on transcriptional control and the constitutive ectopic expression of resistance genes to engineer disease resistance [49]. Unfortunately, engineered resistance often comes with associated fitness costs, making the resulting germplasm undesirable for agricultural applications. Recent studies have highlighted translation as a crucial layer of immune control that enables more precise, pathogen-inducible expression of defense proteins [14,15,28].

Here, we showed that the 5ʹ UTR of *PI4KIII β1* regulates the production of different isoforms of PI4KIII β1 with divergent subcellular localizations. Moreover, we determined that the 5ʹ UTR of *PI4KIII β1* plays an important role in regulating the translational shift by controlling start codon selection. Taking advantage of this property, we engineered the *PI4KIII β1* 5ʹ UTR to specifically induce resistance protein production following the perception of PAMPs, an early step in plant–pathogen interactions. Importantly, transgenic plants expressing the cell death–inducing protein HIR1 via this cassette showed wild type–like growth and enhanced resistance to diverse pathogens belonging to different kingdoms (bacteria, fungi, viruses) and with different lifestyles (from necrotrophic to biotrophic) with no obvious fitness costs. These findings uncover translational regulation as a promising strategy for engineering disease-resistant crops.

## 4 Materials and methods

### 4.1 Plasmid construction

*AtPI4KIII β1*, *5*ʹ *UTR*^*1-45*^*AtPI4KIII β1*, *5*ʹ *UTR*^*1-86*^*AtPI4KIII β1*, and *5*ʹ *UTR*^*1-576*^*AtPI4KIII β1* were individually cloned into the pCHF3-GFP vector. *HIR1*, *GFP*, *GFP-NLS*, *NmM-GFP*, *NmM-GFP-NLS*, *NmM*_*G2A*_*-GFP-NLS*, _*MP*_*NmM-GFP-NLS*, *5*ʹ *UTR*_*MP*_*NmM-GFP-NLS*, *5*ʹ *UTR*_*MP(-C)*_*NmM-GFP-NLS*, *5*ʹ *UTR*_*MP-7His*_*NmM-GFP-NLS*, *5*ʹ *UTR*_*MP(-C)-7His*_*NmM-GFP-NLS*, *5*ʹ *UTR*_*Kozak-MP*_*NmM-GFP-NLS*, *5*ʹ *UTR*_*Kozak-MP(+T)*_*NmM-GFP-NLS*, *5*ʹ *UTR*_*Kozak-MP-5His*_*NmM-GFP-NLS*, *5*ʹ *UTR*_*Kozak-MP(+T)-5His*_*NmM-GFP-NLS*, *5*ʹ *UTR*_*MP-7His*_*HIR1-GFP*, *5*ʹ *UTR*_*MP(-C)-7His*_*HIR1-GFP*, *5*ʹ *UTR*_*Kozak-MP*_*HIR1-GFP*, *5*ʹ *UTR*_*Kozak-MP(+T)*_*HIR1-GFP*, *5*ʹ *UTR*_*Kozak-MP-5His*_*HIR1-GFP*, and *5*ʹ *UTR*_*Kozak-MP(+T)-5His*_*HIR1-GFP* were individually cloned into the pCambia vector. *AtPI4KIII β1pro::*_*MP-7His*_*PI4KIII-GFP* and *AtPI4KIII β1pro::*_*MP-7His(-C)*_*PI4KIII-GFP* were individually cloned into the pBinplus vector. All cDNAs were PCR-amplified using KOD-Plus-Neo High-Fidelity DNA Polymerase (TOYOBO). The resulting PCR fragments were individually cloned into the destination vectors using a One-Step Cloning Kit (Vazyme). All primers used for plasmid construction are listed in Table S1.

### 4.2 Plant material and growth conditions

Transgenic *Nicotiana benthamiana* plants expressing the nuclear marker H2B-RFP (full-length red fluorescent protein fused to the C terminus of histone 2B) were kindly provided by Dr. Michael M. Goodin (University of Kentucky, USA). *N. benthamiana* plants were grown in a growth chamber at 26 °C under a 16-h light/8-h dark photoperiod. The *Arabidopsis* accession Columbia-0 (Col-0) was used as the wild type to generate transgenic plants and for phenotypic comparisons. *Arabidopsis* seeds were sown in trays filled with commercially sterilized peat. Upon full cotyledon development, the plants were cultivated in pots containing commercially sterilized peat and vermiculite at a 2:1 ratio (v:v). The methods for seed sterilization and germination for plant growth were described previously [50].

### 4.3 Generation of transgenic plants

Transgenic *Arabidopsis* plants were generated using the floral dip method [51,52]. Transgenic seeds were selected on standard Murashige and Skoog (MS) medium containing hygromycin (10 mg/L). Antibiotic-resistant transformed plants were verified by PCR using the primers used to generate the expression construct.

### 4.4 Immunoblot analysis

Agroinfiltrated leaf tissue or virus-infected tissue was harvested and homogenized in native extraction buffer (50 mM Tris-HCl, pH 8.0, 0.5 M sucrose, 1 mM MgCl_2_, 10 mM EDTA, 5 mM dithiothreitol) at a ratio of 100 mg tissue per 200 μL buffer. The extracts were centrifuged at 12,000 *g* for 15 min at 4 °C, separated by 12.5% SDS-PAGE, and transferred to nitrocellulose membranes. The membranes were probed with a commercial primary antibody overnight at 4 °C, incubated with a commercial secondary antibody conjugated to horseradish peroxidase, and incubated in ECL (Enhanced chemiluminescence) solution prior to detection.

### 4.5 Agroinfection assays in *Nicotiana benthamiana*

The constructs used for transient expression in *N. benthamiana* leaves were introduced into *Agrobacterium tumefaciens* (strain EHA105) by electroporation. The transformed bacterial cultures were grown individually until reaching an OD_600_ of 0.5–0.8. The cultures were then collected and resuspended in induction buffer (10 mM MgCl_2_, 100 mM MES [pH 5.7], 2 mM acetosyringone) for 3 h at room temperature. The suspensions were adjusted to OD_600_ = 1.0 before leaf infiltration. The suspensions were infiltrated into the leaves of 4- to 6-week-old *N. benthamiana* plants using a 1-mL needleless syringe.

### 4.6 Nuclear–cytoplasmic fractionation assay

Nuclear–cytoplasmic fractionation assays were performed as described previously [53,54]. Plant tissues (0.5 g) were harvested, ground into a fine powder in liquid nitrogen, and mixed with 2 mL/g of lysis buffer (20 mM Tris-HCl, pH 7.5, 20 mM KCl, 2 mM EDTA, 2.5 mM MgCl_2_, 25% glycerol, 250 mM sucrose, and 5 mM DTT) supplemented with protease inhibitor cocktail. The homogenate was filtered through two layers of Miracloth. The flow-through was centrifuged at 1500 *g* for 10 min, and the supernatant, consisting of the cytoplasmic fraction, was centrifuged at 10,000 *g* for 10 min at 4 °C and collected. The pellet was washed four times with 5 mL of nuclear resuspension buffer NRBT (20 mM Tris-HCl, pH 7.4, 25% glycerol, 2.5 mM MgCl_2_, and 0.2% Triton X-100) and resuspended in 500 mL of NRB2 (20 mM Tris-HCl, pH 7.5, 0.25 M sucrose, 10 mM MgCl_2_, 0.5% Triton X-100, and 5 mM β-mercaptoethanol) supplemented with protease inhibitor cocktail (MedChemExpress) and carefully overlaid on top of 500 mL NRB3 (20 mM Tris-HCl, pH 7.5, 1.7 M sucrose, 10 mM MgCl_2_, 0.5% Triton X-100, and 5 mM β-mercaptoethanol) supplemented with protease inhibitor cocktail. The sample was centrifuged at 16,000 *g* for 45 min at 4 °C. The final nuclear pellet was resuspended in 400 μL lysis buffer. As controls for fractionation, actin protein was detected and used as the cytoplasmic marker, and histone H3 was probed and used as the nuclear marker.

### 4.7 Evaluation of disease severity index

Disease severity index (DSI) was evaluated using a scale from 0 (no necrotic spots) to 5 (severe necrosis) at 45 days post-germination (dpg). The fitness cost mediated by immune auto-activation in transgenic *Arabidopsis* plants was evaluated in leaves using DSI where 0 = no visible necrotic spots; 1 = less than 10% of leaf surface with sporadic necrotic spots; 2 = approximately one-quarter of leaf surface with necrotic spots; 3 = approximately one-third of leaf surface with continuous necrotic spots; 4 = approximately two-thirds of leaf surface with continuous necrotic spots; 5 = more than two-thirds of leaf surface with continuous necrotic spots.

### 4.8 Bacterial infection assays

Bacterial infection assays were performed essentially as described previously [40]. Four-week-old *Arabidopsis* plants grown under short-day conditions were infiltrated with an inoculum of *Pseudomonas syringae* pv. *tomato* (OD_600_ = 0.0002 in 10 mM MgCl_2_) using a needleless syringe and kept covered for 24 h. Bacterial growth was determined 3 days after inoculation by plating 1:10 serial dilutions of leaf extract, incubating the plates at 28 °C for 2 days, and counting bacterial colony-forming units (cfu).

### 4.9 Fungal infection assays

Fungal infection assays were performed essentially as described previously [55]. *Botrytis cinerea* strain B05.10, a benomyl-resistant derivative of strain SAS56 [56], was used for the fungal infection assays. Mycelial plugs (5 mm diameter) were inoculated on PDA plates and incubated at 25 °C under a 16-h light/8-h dark photoperiod. After 12 days of incubation, the spores were collected from the plates by washing with 20 mL ddH_2_O per plate. The conidial suspension was filtered through lens-wiping paper and centrifuged at 1200 *g* for 5 min. The conidial pellets were resuspended in 5 mL ddH_2_O, and the conidia were counted using a hemocytometer. Leaves of wild-type and transformed *Arabidopsis* plants were inoculated with 10 μL conidial suspension (1.0 × 10^6^ per mL) and incubated at 25 °C under a 16-h light/8-h dark photoperiod at 90% relative humidity. Lesions were photographed and their sizes measured 3 days after incubation at 25 °C. There were 10 replicates per plant. The experiment was performed at least three times.

### 4.10 Viral infection assays

Viral infection assays were performed as described previously [57]. For CaLCuV infection, 6- to 7-week-old *Arabidopsis* plants were inoculated with *A. tumefaciens* (strain GV3101) harboring two pUC19-based plasmids containing partial tandem copies of CaLCuV DNA-A or DNA-B, respectively. Photographs of symptom development induced by CaLCuV were taken at 14 dpi.

### 4.11 Co-localization analysis

Confocal micrographs were taken under a ZEN LSM780 laser scanning microscope, and images were acquired with ZEN 2012 (version 1.1.13064.302) software. The confocal images were opened in ImageJ software with the Coloc 2 plugin, and image channels were separated using the Coloc 2 plugin. The Pearson value Rcoloc was calculated in both channels using ImageJ software with the Coloc 2 plugin.

### 4.12 Reverse transcription quantitative PCR

Reverse transcription quantitative PCR was performed as described previously [54]. Total RNA was extracted from *A*. *thaliana* leaf tissues using TRIzol reagent (Invitrogen) according to the manufacturer's instructions. First strand synthesis was performed using ReverTra Ace qPCR RT Master Mix with gDNA Remover (TOYOBO). Real-time PCR was conducted by using SYBR Green I Master (Roche) according to the manufacturer's instructions. Primers were used for the amplification of the targets, and the efficiencies of all primers were verified by normal RT-RCR, gel electrophogenesis and melting curve analysis. Primer sequences are listed in Table S1. Expression of *actin* gene was used as internal control.

### 4.13 Prediction of N-myristoylation motif and chloroplast transit peptide

The annotated *Arabidopsis* proteins (35,386) were downloaded from http://www.arabidopsis. org/download files/Proteins/TAIR10 protein lists/TAIR10 pep 20101214. Proteins with chloroplast transit peptide (cTP) (3574) were selected for further analysis. Proteins with both a cTP and an N-myristoylation motif encoded by a gene with an in-frame start codon (14) were selected for functional analyses. The cTP and the N-terminal myristoylation motif were predicted by SignalP and Expasy Myristoylator software, respectively.

### 4.14 Accession numbers

Gene sequences are available in The Arabidopsis Information Resource or Sol Genomics Network under accession numbers AT5G64070 (AtPI4KIII β1) and Niben044Scf00010885 (NbHIR1).

### 4.15 Statistical analysis

All statistical analyses were performed using GraphPad Prism 8.0 software. A two-sided, unpaired Student's *t*-test was performed. Details about statistical analysis are provided in the figure legends. Data are represented as mean ± SD as indicated.

## CRediT authorship contribution statement

**Yuzhen Mei:** Writing – original draft, Validation, Methodology, Investigation, Formal analysis, Data curation. **Xiaofei Cheng:** Writing – review & editing, Formal analysis. **Yaqin Wang:** Investigation, Formal analysis. **Fangfang Li:** Writing – review & editing, Investigation. **Xueping Zhou:** Writing – review & editing, Supervision, Project administration, Funding acquisition, Conceptualization.

## Declaration of competing interest

The authors declare no competing interests.

## Data Availability

The data that support the findings of this study are available from the corresponding author upon reasonable request.
