# Supplementary material for: A pathogen-induced translational shift enhances plant disease resistance without obvious fitness costs
Source: aBIOTECH. 2026 Feb 3;7(2):100025. doi: 10.1016/j.abiote.2026.100025 (PMC12973399; doi:10.1016/j.abiote.2026.100025)
Supplement: Multimedia component 1 [file mmc1.pdf]

Supporting Information

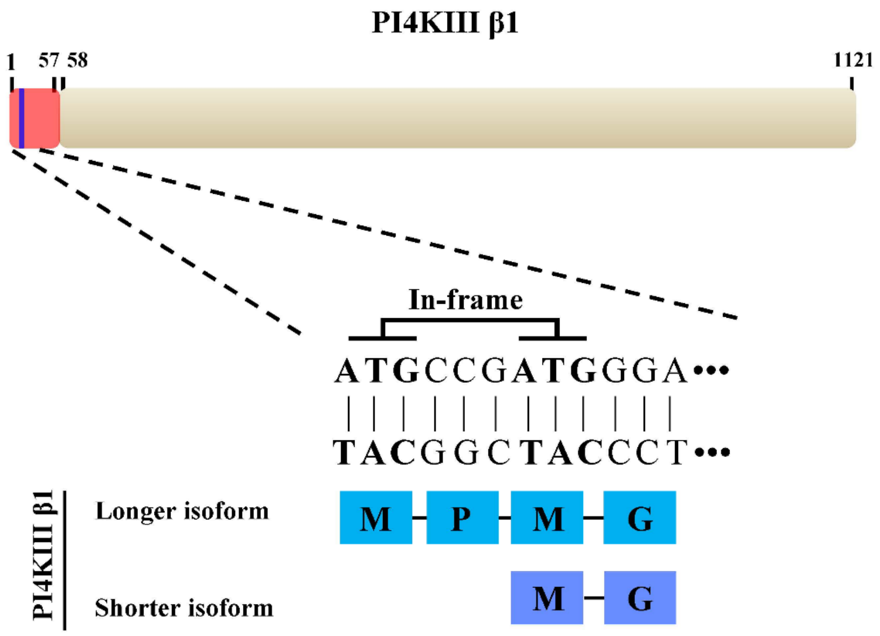

Fig. S1 Schematic representation of chloroplast transit prptide (cTP) predicted by the SignalP software in AtPI4KIII β1. Red rectangle indicates the cTP of AtPI4KIII β1. Blue rectangle represents the N-myristoylation motif of AtPI4KIII β1. Lower panel indicates nucleotide sequence of Arabidopsis *PI4KIII β1* containing two potential in-frame start codons. The encoded amino acid sequences of shorter and longer isoforms of AtPI4KIII β1 are shown.

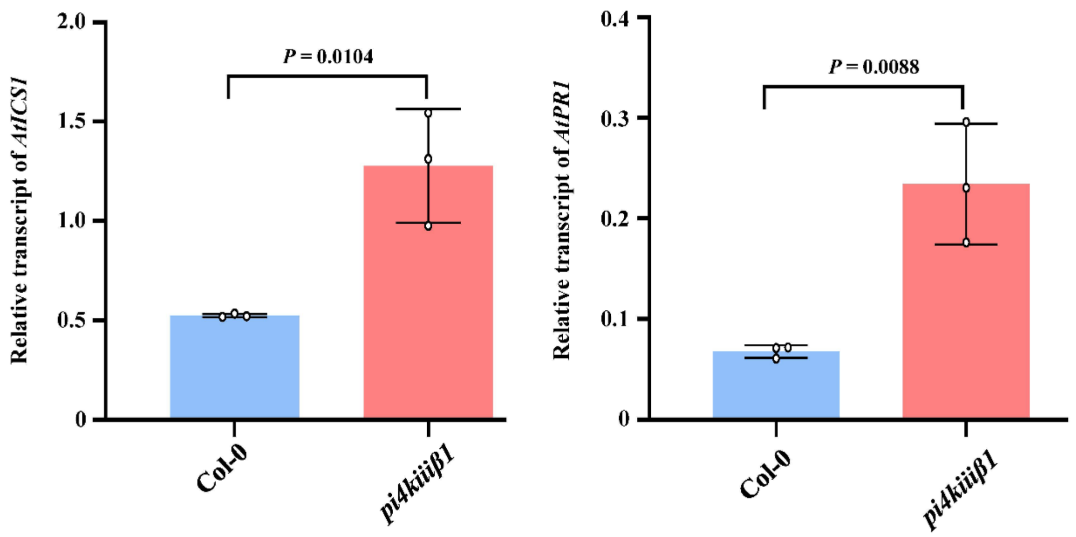

Fig. S2 Identification of PI4KIII β1 functions in plant immunity regulation. Quantitative PCR analysis of *ICS1* and *PR1* expression in Col-0 and *pi4kiii β1* mutant. *Actin2* was used as the normalizer. Statistical differences were analyzed by two-sided, unpaired Student's t-test. Data are mean ± SD of three independent biological replicates. Individual *P* values are denoted above the comparison lines.

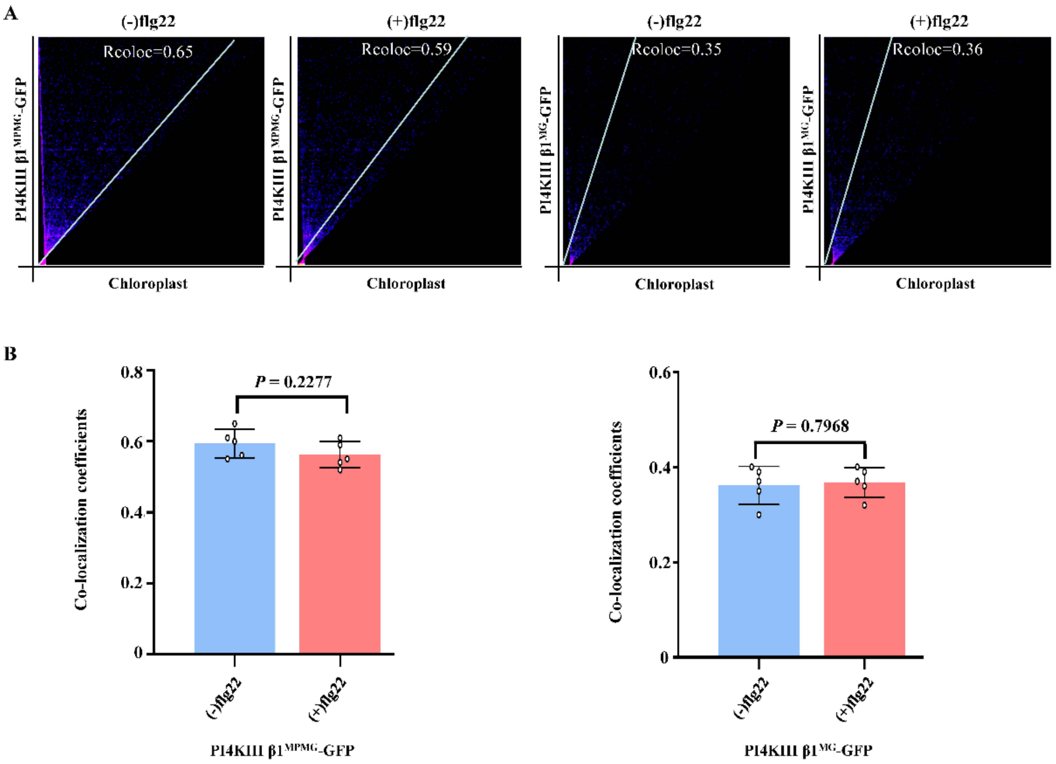

Fig. S3 Co-localization analysis of the two PI4KIII β1 variants fused to GFP with chloroplast in epidermal cells of *Nicotiana benthamiana* plants untreated or treated with flg22. A The Pearson value Rcoloc was introduced into co-localization analysis. The Pearson value Rcoloc was calculated using ImageJ software with the Coloc 2 plugin. **B** Graph indicates the co-localization coefficients (n = 5, ±SEM) of two PI4KIII β1 variants and chloroplasts in **Fig. 1D.** Statistical analyses were performed using two-sided, unpaired Student's t-test. Individual P values are denoted above the comparison lines.

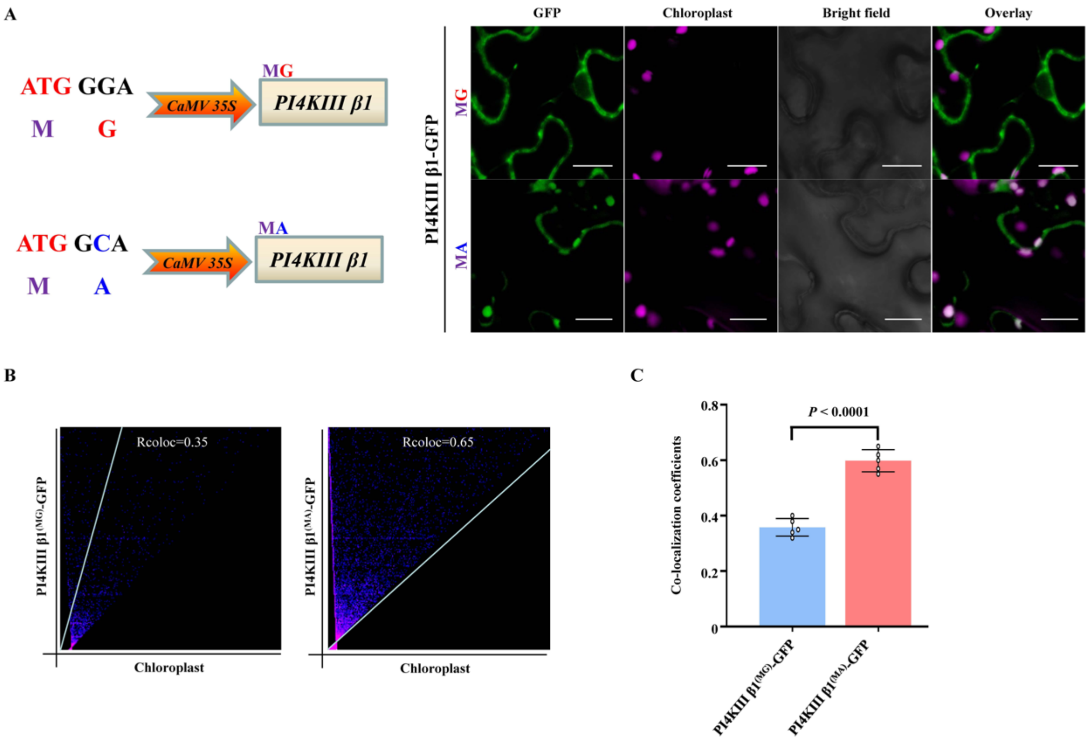

Fig. S4 N-myristoylation of shorter PI4KIII β1 variant overrides its chloroplast transit peptide (cTP) function. A Nucleotide sequences and subcellular localizations of the shorter *PI4KIII β1* variant and its non-myristoylatable mutant. The encoded amino acid sequence of shorter PI4KIII β1 variant and PI4KIII $\beta1^{G2A}$ mutant are shown. Subcellular localizations of PI4KIII β1 variant and its non-myristoylatable mutant fused to GFP in epidermal cells of *Nicotiana benthamiana* plants. Scale bar = 50 μm. **B** Co-localization analysis of the shorter PI4KIII β1 variant and PI4KIII $\beta1^{G2A}$ mutant fused to GFP with chloroplast in epidermal cells of *N. benthamiana* plants. The Pearson value Rcoloc was calculated using ImageJ software with the Coloc 2 plugin. **C** Graph indicates the co-localization coefficients (n = 5, ±SEM) of shorter PI4KIII β1 variant or PI4KIII $\beta1^{G2A}$ mutant and chloroplasts in **Fig. S4A**. Statistical analyses were performed using two-sided, unpaired Student's t-test. Individual *P* values are denoted above the comparison lines.

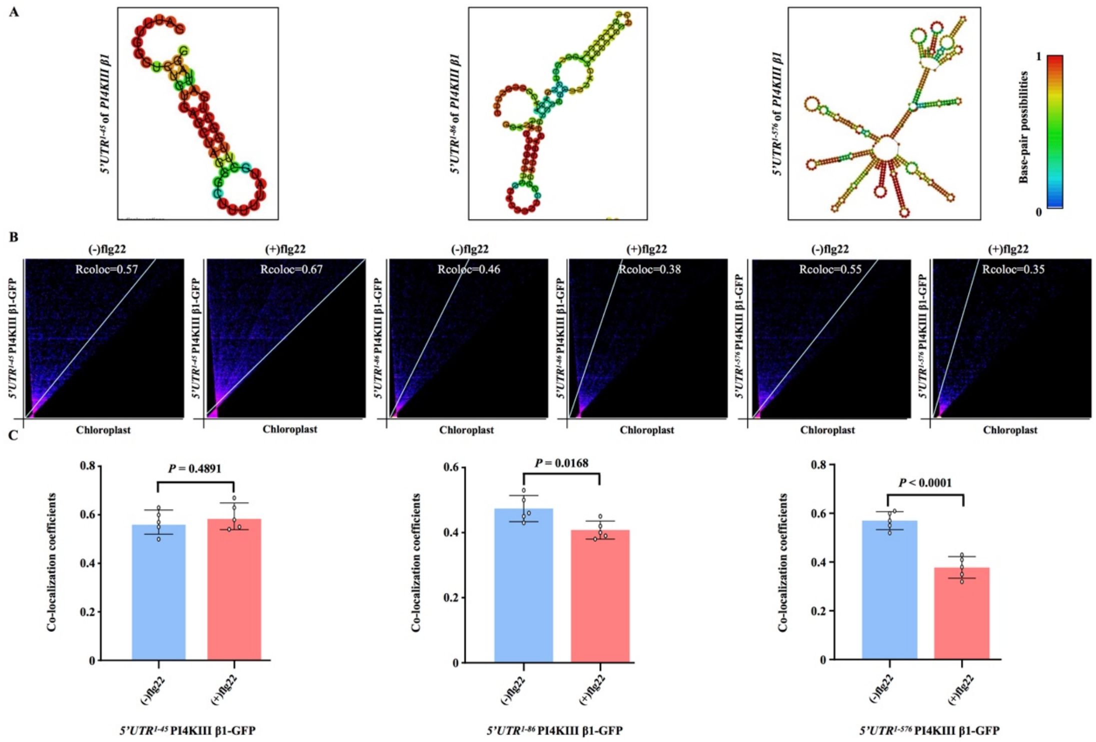

Fig. S5 5' UTR of *PI4KIII β1* plays an important role in translation shift upon PAMP treatment. **A** Predicted RNA secondary structure of full-length 5' UTR of *PI4KIII I4* and two truncated versions by RNAfold software. The predicted structure is colored by base-pairing probabilities. **B** Co-localization analysis of the PI4KIII β1 driven by different lengths of 5' UTR of *PI4KIII β1* with chloroplast in epidermal cells of *N. benthamiana* plants untreated or treated with flg22. The Pearson value Rcoloc was calculated using ImageJ software with the Coloc 2 plugin. **C** Graph indicates the co-localization coefficients (n = 5, ±SEM) of PI4KIII β1-GFP driven by different lengths of 5' UTR of *PI4KIII β1* and chloroplasts in **Fig. 1E**. Statistical analyses were performed using two-sided, unpaired Student's t-test. Individual *P* values are denoted above the comparison lines.

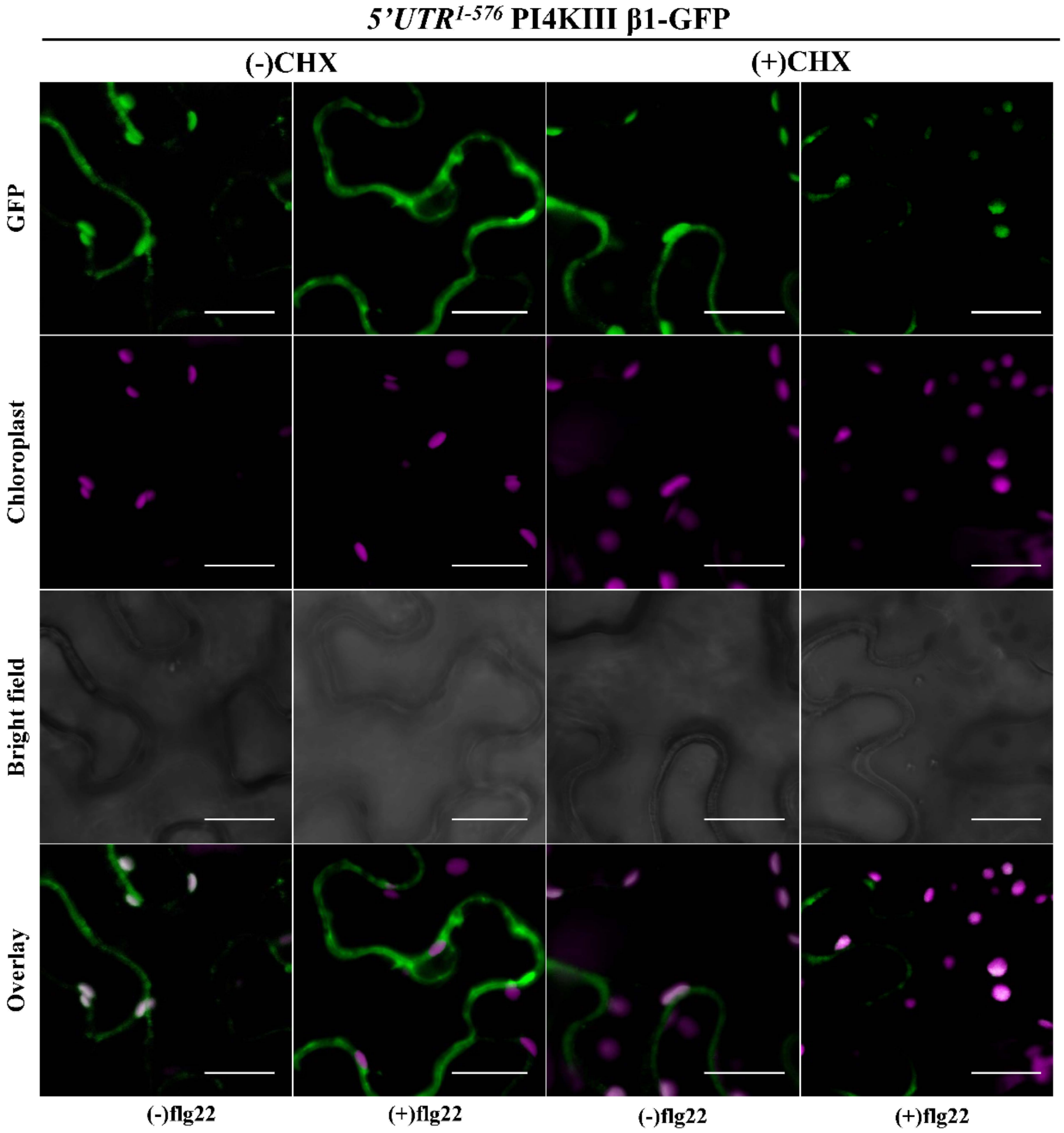

Fig. S6 Subcellular localization of PI4KIII β1-GFP driven by CaMV *35S* promoter and *PI4KIII β1* full-length 5' UTR following treatment with 1 μM flg22 (24 h post-treatment) in the presence of CHX (50 mg/mL, 2 h prior to flg22 treatment). Scale bar = 50 μm.

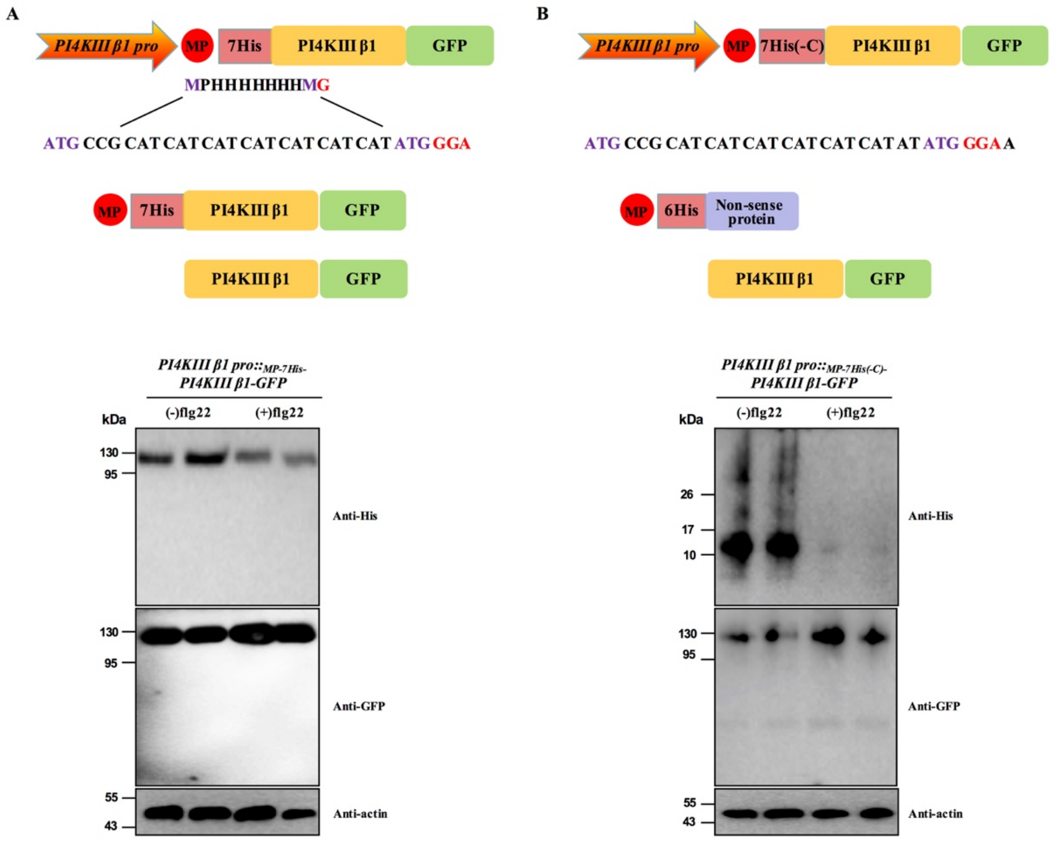

Fig. S7 Analysis of the translation shift by native *PI4KIII β1* promoter upon flg22 treatment.

A-B Immunoblot analysis of the alternative AUG usage under the *PI4KIII β1* native promoter upon flg22 treatment. Immunoblot analysis was conducted with the antibodies specific to the indicated proteins. Leaves of *Nicotiana benthamiana* plants transiently expressing indicated proteins were treated with 1 μM flg22 (24 h post-treatment).

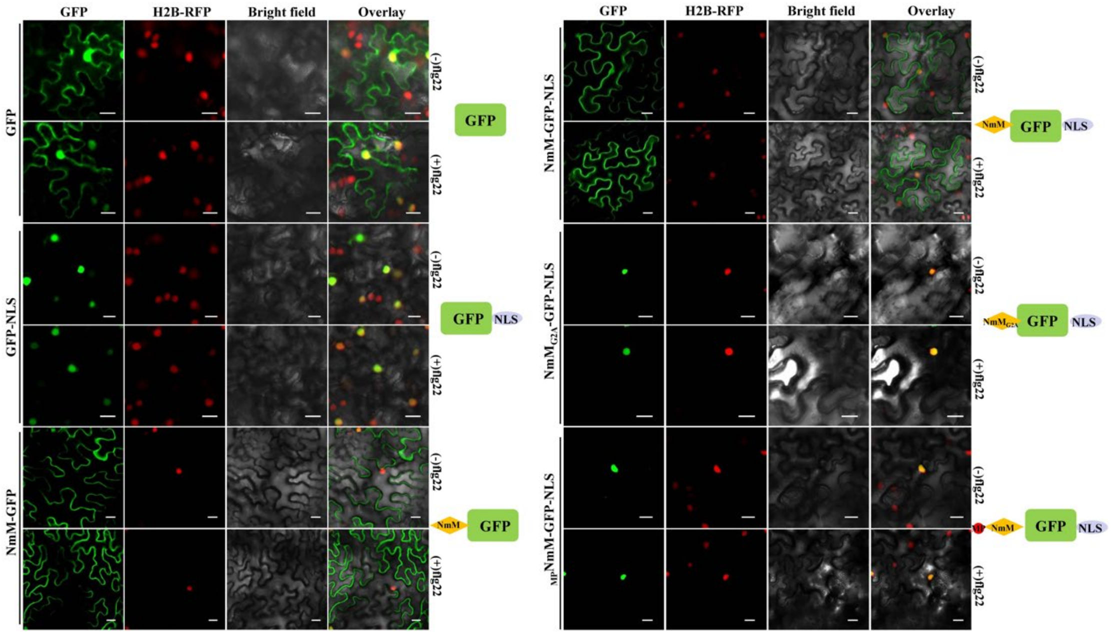

Fig. S8 Identification of the cellular distribution of GFP fused to specific signal sequences in epidermal cells of transgenic H2B-RFP *Nicotiana benthamiana* plants. NLS, nuclear localization signal. NmM, the peptide derived from putative plant myristoylated protein AtCDPK1 contains a conserved N-myristoylation motif. NmM$_{G2A}$ is the non-myristoylable mutant of NmM.

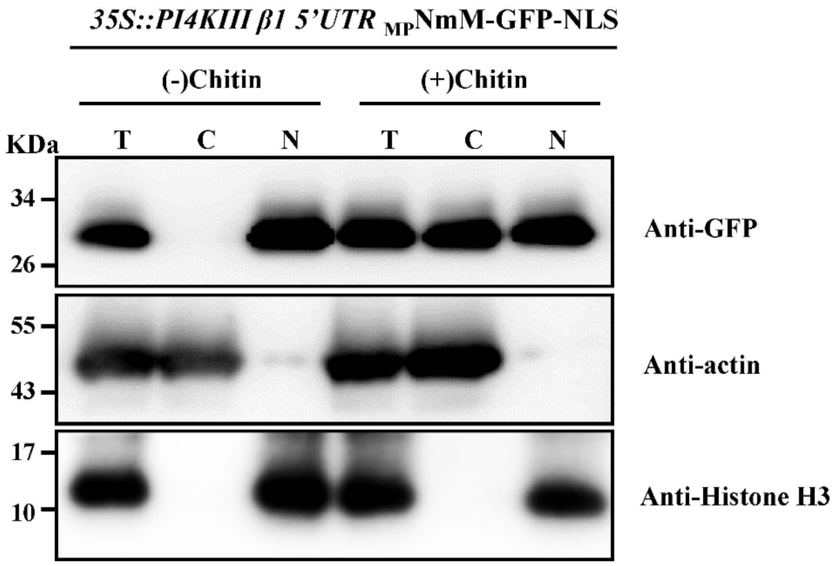

Fig. S9 Nuclear-cytoplasmic fractionation analysis of the altered cellular distribution of $_{MP}$NmM-GFP-NLS in the absence or presence of chitin (10 μg/mL). Western blot analysis was conducted with antibodies specific to the indicated proteins. NbActin was used as a marker for the cytoplasmic fraction and histone H3 was used as a marker for the nuclear fraction. T, C, and N indicate total, cytoplasmic, and nuclear extracts, respectively.

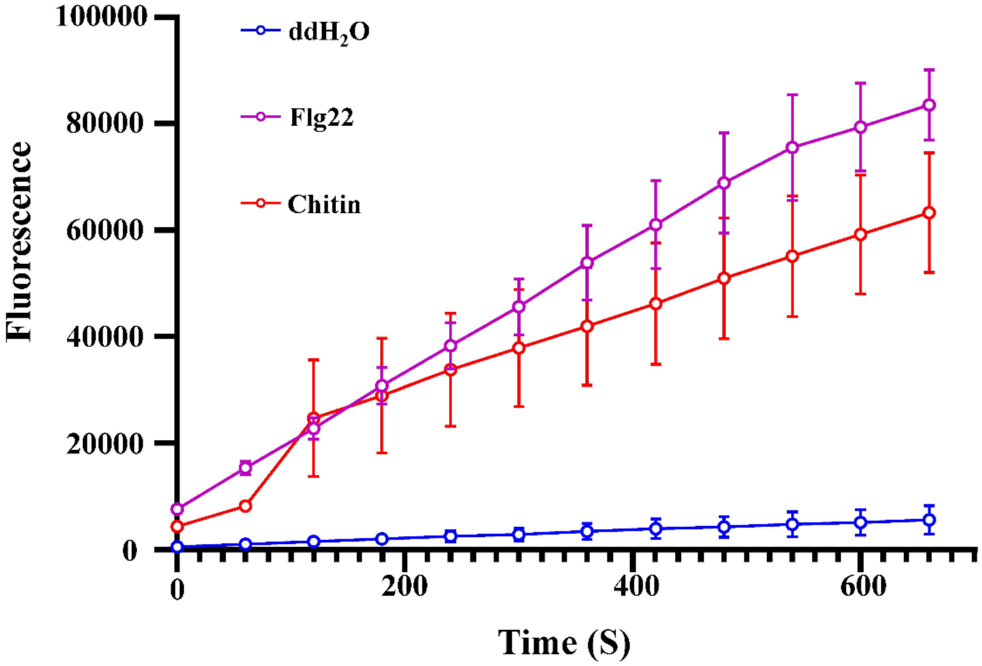

Fig. S10 Detection of the target gene expression through translation shift by *PI4KIII β1* 5' UTR upon different pathogen-associated molecular patterns (PAMPs) treatment. Leaf disc of the transgenic *A. thaliana* plants harboring the coding sequence of $_{\text{Kozak-}\mathit{MP(+T)\text{-}5His}}$*LUC* driven by CaMV *35S* constitutive promoter were treated with ddH_2O, flg22, or chitin for 20 min. Fluorescence is measured every 60 s for 11 min. Error bar denotes the standard deviation of three biological replicates. The blue, purple and red curves indicate the fluorescence of transgenic *A. thaliana* plants treated with ddH_2O, flg22, or chitin respectively.

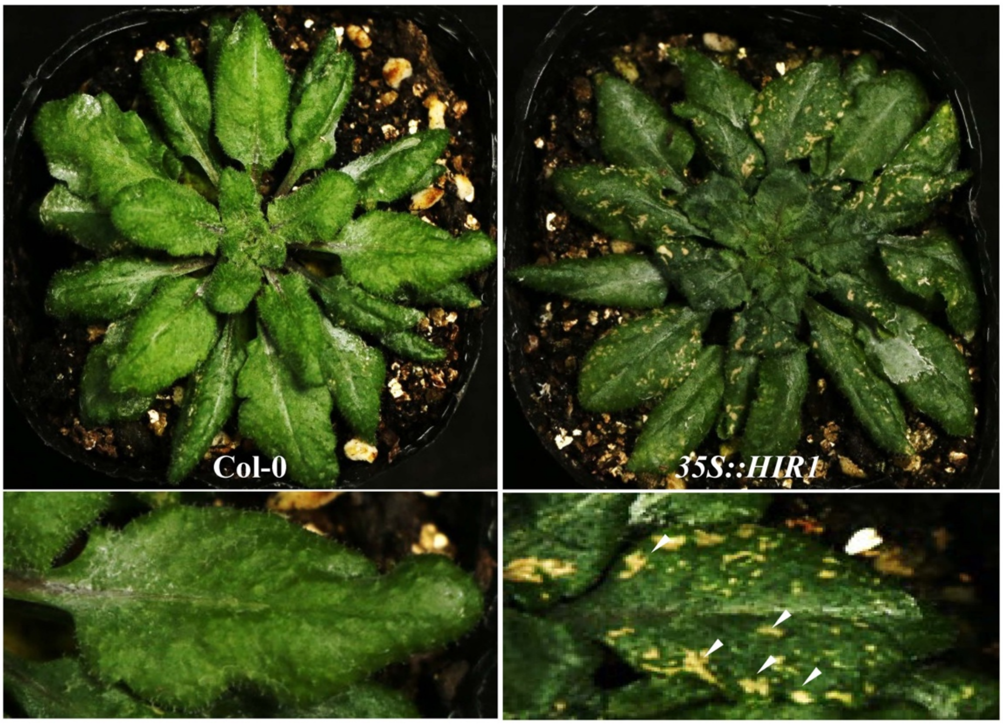

Fig. S11 Developmental phenotypes of wild-type and *35S::HIR1* transgenic *A. thaliana* plants.

Overexpression of HIR1 induces activation of immunity and growth defects. Arrowheads indicate the leaf lesions caused by HIR1 overexpression.

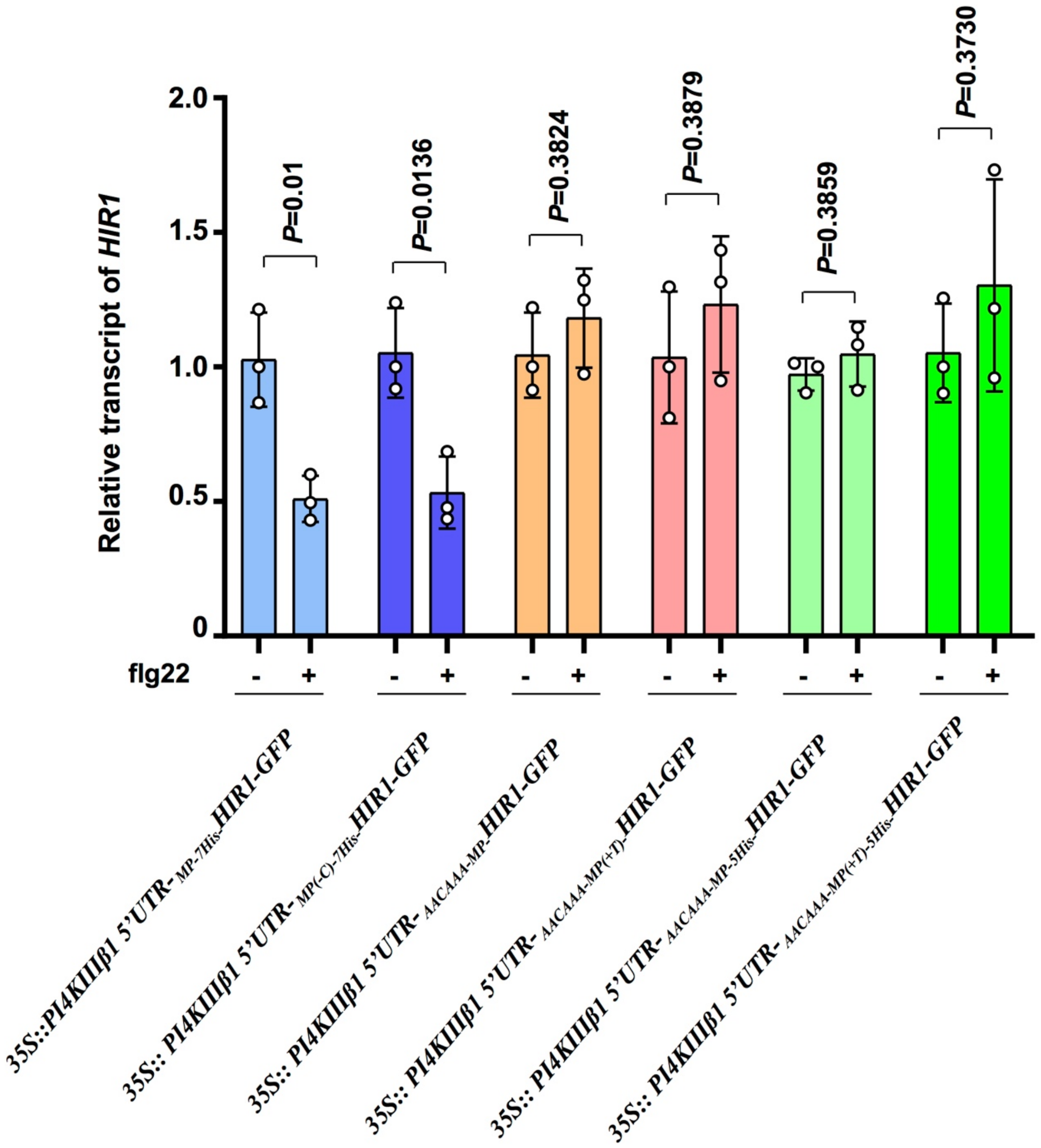

Fig. S12 Quantitative PCR analysis of *HIR1* expression in stable transgenic *Arabidopsis thaliana* plants treated with H_2O or flg22. *Actin2* was used as the normalizer. Statistical differences were analyzed by two-sided, unpaired Student's t-test. Data are mean ± SD of three independent biological replicates. Individual *P* values are denoted above the comparison lines.

Table S1 Primers used in study

Gene	Sequence
**Cloning primers**	
pCambia-GFP-F-SalI	ATCTCGAGGCGCGCCGTCGACATGGTGAGCAAGGGCGAGGAG
pCambia-GFP-R-SalI	GTCGTCCTTGTAGTCCATGTCGACTTACTTGTACAGCTCGTCCATGCC
pCambia-GFP-NLS-R-SalI	GTCGTCCTTGTAGTCCATGTCGACTTAAACCTTCCTCTTCTTCTTAGGAACCTTCCTCTTCTTCTTAGGCTTGTACAGCTCGTCCAT
pCambia-NmM-GFP-F	CTCGAGGCGCGCCGATGGGCGGTTGCTTCTCCAAGAAATATAGAAGGGTGAGCAAGGGC
pCambia-NmM-GFP-NLS-F1	ATCTCGAGGCGCGCCGATGGGAGGTTGCTTCTCCAAGAAATATAGAAGGGTGAGCAAGGGC
pCambia-NmM-GFP-NLS-F2	ATGGGCGGTTGCTTCTCCAAGAAATATAGAAGGGTGAGCAAGGGCGAGGAGCTGTTC
pCambia-NmM$_{G2A}$-GFP-NLS-F	ATCTCGAGGCGCGCCGATGGCCGGTTGCTTCTCCAAGAAATATAGAAGGGTGAGCAAGGGC
pCambia-$_{MP}$NmM-GFP-NLS	ATCTCGAGGCGCGCCGATGCCGATGGGAGGTTGCTTCTCCAAGAAATATAGAAGGGTGAGCAAGGGC
pCambia-PI4KIII 5'UTR-F1	ATCTCGAGCCGCGCCGTCGACTTAAAAGTATTGCGAACGATAATTAC
pCambia-PI4KIII 5'UTR-$_{MP}$NmM-R1	TTTCTTGGAGAAGCAACCGCCCATCGGCATCTTAATCAGCCAAGCATAAAAAGC
pCambia-PI4KIII 5'UTR-$_{MP(-C)}$ NmM-R2	TTTCTTGGAGAAGCAACCGCCCATCGCATCTTAATCAGCCAAGCATAAAAAGC
pCambia-PI4KIII 5'UTR-$_{MP\text{-}7His\text{-}}$NmM-R3	GGAGAAGCAACCGCCCATATGATGATGATGATGATGATGCGGCATCCTAATCAGCCAAG
pCambia-PI4KIII5'UTR-$_{MP(-C)\text{-}7His\text{-}}$NmM-R4	GGAGAAGCAACCGCCCATATGATGATGATGATGATGATGCGCATCCTAATCAGCCAAG
pCambia-PI4KIII5'UTR-$_{AACAAA\text{-}MP\text{-}}$NmM-R5	TTTCTTGGAGAAGCAACCGCCCATCGGCATTTTGTTCCTAATCAGCCAAGCATAAAAAG
pCambia-PI4KIII5'UTR-$_{AACAAA\text{-}MP(+T)\text{-}}$NmM-R6	TTTCTTGGAGAAGCAACCGCCCATACGGCATTTTGTTCCTAATCAGCCAAGCATAAAAAG
pCambia-PI4KIII5'UTR-$_{AACAAA\text{-}MP\text{-}5His\text{-}}$NmM-R7	GGAGAAGCAACCGCCCATATGATGATGATGATGCGGCATTTTGTTCCTAATCAGCCAAAG
pCambia-PI4KIII5'UTR-$_{AACAAA\text{-}MP(+T)\text{-}5His\text{-}}$NmM-R8	GGAGAAGCAACCGCCCATATGATGATGATGATGACGGCATTTTGTTCCTAATCAGCCAAAG
pBinplus-PI4KIIIpro-F1-SalI	CCAAGCTTGCATGCCTGCAGGTCGACGTACAAAATTTCTTCATCTGAAAA
pBinplus-PI4KIIIpro-$_{MP\text{-}7His\text{-}}$PI4KIII-F2	TTATGCTTGGCTGATTAGGATGCCGCATCATCATCATCATCATCATATGGGAATGCCGATGGGACGCT

pBinplus-PI4KIIIpro-MP-7His(-C)-PI4KIII-F3	TTATGCTTGGCTGATTAGGATGCCGCATCATCATCATCATCATATATGGGAATGCCGATGGGACGCT
pBinplus-PI4KIIIpro-GFP-R1-EcoRI	GTGAATTGTTAATTAAGAATTC
pBinplus-PI4KIIIpro-MP-7His-PI4KIII-R2	AGCGTCCCATCGGCATTCCCATATGATGATGATGATGATGATGCGGCATCCTAATCAGCCAAGCATAA
pBinplus-PI4KIIIpro-MP-7His(-C)-PI4KIII-R3	AGCGTCCCATCGGCATTCCCATATATGATGATGATGATGATGCGGCATCCTAATCAGCCAAGCATAA
pCHF3-HIR1-GFP-F-KpnI	GGGGACGAGCTCGGTACCATGTGGACATGTTGTGGATTAA
pCHF3-HIR1-GFP-F-BamHI	GCCCTTGCTCACCATGGATCCATGCTCAATAGAAGCCTGAAGAAG
pCHF3-PI4KIII5'UTR-MP-7His-HIR1-GFP-F	ATGCTTGGCTGATTAGGATGCCGCATCATCATCATCATCATCATATGTGGACATGTTGT
pCHF3-PI4KIII5'UTR-MP(-C)-7His-HIR1-GFP-F	ATGCTTGGCTGATTAGGATGCGCATCATCATCATCATCATCATATGTGGACATGTTGT
pCHF3-PI4KIII5'UTR-AACAAA-MP-HIR1-GFP-F	ATGCTTGGCTGATTAGGAACAAAATGCCGATGTGGACATGTTGTGGATTA
pCHF3-PI4KIII5'UTR-AACAAA-MP(+T)-HIR1-GFP-F	ATGCTTGGCTGATTAGGAACAAAATGCCGTATGTGGACATGTTGTGGATTA
pCHF3-PI4KIII5'UTR-AACAAA-MP-5His-HIR1-GFP-F	ATGCTTGGCTGATTAGGAACAAAATGCCGCATCATCATCATCATATGTGGACATGTTGTGGATTA
pCHF3-PI4KIII5'UTR-AACAAA-MP(+T)-5His-HIR1-GFP-F	ATGCTTGGCTGATTAGGAACAAAATGCCGTCATCATCATCATCATATGTGGACATGTTGTGGATTA
pCHF3-PI4KIII5'UTR-HIR1-GFP-R	TCCTCGCCCTTGCTCACCATTTAATGCTCAATAGAAGCCTGAAGAAG
pCHF3-AtPI4KIII-GFP-F-KpnI	GGGGACGAGCTCGGTACCATGCCGATGGGACGCTTTCTATCTTTGG
AtPI4KIII5'UTR-F1(-1--45)	GATTTGGGTCTGTGAGCTAGGGCTTTTTATGCTTGGCTGATTAGGATGCCGATGGGACG
AtPI4KIII5'UTR-F1(-1--86)	GTTTGTTGGAAGAGTCTCCTGGATCTGTTGTCTCGACGGATGATTTGGGTCTGTGAGCT
pCHF3-AtPI4KIII(G2)-GFP-F-KpnI	GGGGACGAGCTCGGTACCATGGGACGCTTTCTATCTTTGG
pCHF3-AtPI4KIII-GFP-R-BamHI	GCCCTTGCTCACCATGGATCCCAATATTCCATTTAAGACCCGTTGGTAGTAATC
AtPR1-qPCR-F	CACAACCAGGCACGAGGAGC
AtPR1-qPCR-R	CACCTCACTTTGGCACATCCG
AtICS1-qPCR-F	GCAATTGATTGGCTTCAAGCCCAG
AtICS1-qPCR-R	GGTTCCCATTCAACAGCGATCTTG
AtACT7-qPCR-F	GATTTGGCATCACACTTTCTACAATG
AtACT7-qPCR-R	CAAGACGAAGGATAGCATGAGGAAG
HIR1-qPCR-F	AGTGCAGCAACTAGATGTGCGC

HIR1-qPCR-R	CTCAGCTTCTGCCTTCTCATTAGC
